# Equipping Youth to Think and Act Responsibly: The Effectiveness of the “EQUIP for Educators” Program on Youths’ Self-Serving Cognitive Distortions and School Bullying Perpetration

**DOI:** 10.3390/ejihpe12070060

**Published:** 2022-07-18

**Authors:** Mirella Dragone, Concetta Esposito, Grazia De Angelis, Dario Bacchini

**Affiliations:** Department of Humanistic Studies, University of Naples “Federico II”, 80133 Napoli, Italy; mirella.dragone@unina.it (M.D.); concetta.esposito3@unina.it (C.E.); grazia.deangelis2@gmail.com (G.D.A.)

**Keywords:** EQUIP for educators, bullying perpetration, self-serving cognitive distortions, environmental sensitivity, adolescence

## Abstract

School bullying is a serious public health concern in many countries worldwide. Over recent decades, several effective anti-bullying prevention programs have been developed. This study aimed to evaluate the effectiveness of an adapted version of the “Equipping Youth to Help One Another (EQUIP) for Educators” (EfE) program in reducing adolescents’ engagement in school bullying perpetration by correcting their use of self-serving cognitive distortions (CDs). Furthermore, guided by the vantage sensitivity framework, we investigated whether the intervention effects varied depending on the adolescents’ gender and environmental sensitivity. A quasi-experimental pre-test/post-test with a control group design involved 354 Italian middle and high school adolescents (51.7% males; *M_age_* = 14.86, *SD* = 2.54). Both the control (*n* = 187) and experimental group (*n* = 167) completed self-report questionnaires, before and after the intervention. Structural equation modeling revealed a significant moderated mediation effect: highly sensitive males participating in the EfE program decreased their engagement in bullying by reducing CDs, compared to females and those with low- and medium- sensitivity. These findings support the effectiveness of the EfE program in equipping youth to think and act more responsible and shed light on “why” and “for whom” the intervention might work better to counteract school bullying during adolescence.

## 1. Introduction

School bullying is a subtype of proactive aggression and intentional behavior carried out by a group or an individual repeatedly against a victim who cannot easily defend him or herself [1]. During the school years, bullying is the most common expression of violence in the peer context [2]. Given the systematic nature of power abuse [3] to the detriment of weaker victims, school bullying has been considered a behavior of greater intrinsic moral relevance compared to other aggressive behaviors [4,5,6] and represents one of the most significant social problems affecting children and adolescents in all parts of the world [7].

Although a wide variation has been documented in the prevalence rates of bullying across countries [2], it has been estimated that, globally, almost one in three children experiences school bullying victimization, and one out of four in Europe [8]. From a developmental perspective, the bullying trend is characterized by an initial increase as youths make the transition from elementary to middle school, peak after school transitions, and then a gradual decrease during high school years [9,10,11].

The literature has widely documented the negative consequences, in terms of mental health and educational outcomes, associated with school bullying experiences [2]. Prospective studies found that bullying perpetrators are more likely, over time, to carry weapons (e.g., [12]), to be involved in criminal offending [13,14], or use drugs (e.g., [15,16]) as well as to drop out from school [17].

Given the detrimental effects of school bullying on youths’ adjustment, it is understandable that growing interest has developed over the decades for carrying out effective anti-bullying prevention efforts [18]. Several systematic and meta-analytical reviews (e.g., [18,19]) highlighted the effectiveness of some school-based programs in reducing bullying perpetration and victimization [1,20,21,22,23]. However, given the high variability in the effectiveness of these programs, it has been suggested by Smith et al. [24] to pay greater attention in exploring the principles of “what and why, for whom, and under what circumstances” some interventions work.

To date, only a few studies have systematically examined the mediation and moderation mechanisms involved in explaining “why” and “for whom” anti-bullying intervention programs may activate expected behavioral changes. Based on previous research finding moral cognitive processes as key factors for explaining bullying perpetration (e.g., [25,26]) as well as that targeted interventions for aggressive behavior including cognitive behavioral approaches are effective in reducing peer aggression [27], this study aimed to evaluate, in the Italian school context, the effectiveness of an adapted version of the cognitive-behavioral program “EQUIPping Youth to Help One Another (EQUIP) for Educators” (EfE; [28]) in counteracting the bullying perpetration by correcting adolescents’ use of self-serving cognitive distortions. Furthermore, informed by the Vantage Sensitivity framework [29,30], according to which some individuals may be more likely than others to benefit from intervention, we examined whether individual differences in environmental sensitivity could moderate the intervention effects on expected changes. The main novelty of the current study is that it is the first to have adopted the EfE program in the Italian school context and is one of the few (e.g., [31]) to have tested simultaneously the role of gender and environmental sensitivity in moderating adolescents’ response to anti-bullying intervention.

### 1.1. Towards a Cognitive-Behavioral Program for Preventing and Counteracting School-Bullying: The “EQUIP for Educators”

Many prevention efforts designed to reduce antisocial behaviors among youths follow a multicomponent intervention approach targeting the multifaceted needs of behaviorally at-risk youths at the same time [32]. Cognitive-behavioral programs proved to be relatively effective—although significant variations have been found in the effect sizes across studies (e.g., [33,34,35,36]), and the effects of such programs specifically on bullying behavior still warrant further evaluation [27]. Such programs are based on the primary assumption of the cognitive-behavioral approach, according to which dysfunctional thinking patterns contribute to the development and persistence of antisocial behavior. By altering these biased thinking patterns, it would be possible to modify antisocial aspects of personality and consequent behaviors [37,38]. Therefore, cognitive and behavioral changes are assumed to reinforce each other by teaching new skills in areas where at-risk youth show deficits [39]. Among others, some manualized cognitive-behavioral programs applied in juvenile correctional facilities, clinical or school settings, and aimed at counteracting conduct problems or aggressive behaviors, include the Aggression Replacement Training (ART; [40]), the Coping Power Program (CPP; [41]), and the “EQUIPping Youth to Help One Another (EQUIP)” program [42]. These interventions have been informed by theoretical models suggesting that a combination of social-cognitive, emotional, and neuropsychological processes contribute to the development and maintenance of aggressive behaviors in youth. By working on youths’ behavioral and cognitive abilities to recognize and communicate emotions and manage their feelings positively, these programs have yielded positive results in preventing externalizing problem behaviors among children and adolescents (e.g., [43,44]).

More specifically, the “EQUIP for Educators” (EfE; [28]) is one of the promising multicomponent school-based cognitive-behavioral programs which results from an adaptation of the original treatment program “EQUIP” for juvenile offenders [42]. It is dedicated to both primary and secondary prevention in educational contexts. Developed within Gibbs and colleagues’ [45] “three Ds” model, the EfE program aims to equip young people with behavioral problems in thinking and acting more responsibly by targeting three core aspects generally characterizing antisocial youths’ social cognition [42,46,47]: the use of self-serving cognitive Distortions—defined as “inaccurate or biased ways of attending to or conferring meaning upon experiences” [48] (p. 1); social skills Deficiencies—defined as “imbalanced and unconstructive behavior in difficult interpersonal situations” [42] (p. 165), and sociomoral developmental Delay—that is, persistence, during adolescence, of immaturity in moral judgment and egocentric bias. Thus, combining a peer-helping (or mutual-help) and a skills-training (or cognitive behavioral) approach, that is based on the ART curriculum [40], the EfE program can be considered a multicomponent program aimed at: (i) decreasing self-serving cognitive distortions (particularly relating to anger management); (ii) improving social skills; and (iii) stimulating moral judgment development, in the context of a positive peer culture [49].

Based on the social-cognitive approaches (e.g., [50,51]), according to which, people act upon their interpretation of social events [52] and antisocial behavior is based on deficiencies in interpreting these events (i.e., cognitive distortions), at the heart of the EfE psychoeducational curriculum is the correction of “thinking errors” or self-serving cognitive distortions [32] since, as previous studies have demonstrated (e.g., [25,53]), when reaching high levels, they facilitate externalizing problems and some types of aggressive behaviors among peers, such as bullying.

Cognitive distortions have been distinguished by Barriga and Gibbs [54] into primary and secondary, depending on their function. Primary distortions are “self-centered” attitudes, thoughts, and beliefs which reflect more immature moral judgment stages and serve as primary motivators or “pretexts” of aggressive behaviors. Secondary distortions take the form of pre- or post-rationalizations or “excuses” for facilitating aggressive behaviors. Indeed, their function is to overcome dissonance between individual moral standards and behavioral transgressions neutralizing potential empathy and guilt towards the victim, thus avoiding damage to one’s self-image when engaging in misbehaviors [55,56]. Such cognitive rationalizations may assume the form of: (i) blaming others (i.e., “misattribution of blame for victimization or misfortune to innocent others”; [54], p. 334); (ii) minimizing/mislabeling (i.e., antisocial behavior is depicted as not really harmful or even as an admirable outcome); and (iii) assuming the worst (i.e., gratuitous attribution of hostile intentions to others in a social situation; treating the worst scenario as inevitable; believing that improvement of one’s own or others’ behavior is impossible). These distorted thinking patterns are assumed to block moral judgment development because one does not consider oneself to be responsible for one’s antisocial behavior, as those fulfil a defensive or neutralizing role [57].

In line with these considerations, a growing number of research (e.g., [26,58]), developed within the theoretical framework of moral disengagement [50,59], confirmed the key role of moral neutralization processes in promoting and strengthening youths’ aggressive tendencies. However, to date, only a few studies have systematically investigated the predictive role of cognitive distortions, as conceptualized by Gibbs and colleagues [42], in explaining peer-related aggression or bullying behavior [25,37]. Based on the findings of these studies highlighting that youth need to construct attitudes and beliefs that justify their immoral actions in order to maintain a positive self-concept, it might assume that when they become able to correct their “thinking errors” or cognitive distortions, they can refute the rationalizations that block or neutralize their empathy for actual or prospective victims [28], thus reducing the probability to engage in aggressive behavior. Overall, most of the studies on the effects of the EQUIP program seem to indicate that behavioral change is possible after the cognitive change [60].

### 1.2. Empirical Evidence Supporting the Effectiveness of the “EQUIP” Program

The EQUIP was originally developed as a treatment program for juvenile offenders and it has been found to be effective in promoting social skills [61] as well as in reducing recidivism rates after the intervention [61,62,63]. The main focus of the intervention is to decrease the tendency to make self-serving cognitive distortions and to develop less positive attitudes towards antisocial behaviors [47,64].

Based on these promising results, an adapted version of the original treatment program for juvenile offenders [42], was developed (EfE; [28]), taking the form of a psychoeducational prevention curriculum targeting students from the general population.

So far, only a few studies have evaluated the effects of the EfE in the school context and were mainly carried out in Canada, Spain, and the Netherlands. Their results have corroborated the efficacy of the program in equipping students with: (i) skills for managing anger and correcting cognitive distortions [32,38,65] as well as for reducing pro-violence attitudes [38,65]; (ii) social skills for constructive prosocial behavior [32,65]; and (iii) skills for remediating development delay in moral judgment [32]. More specifically, as regards the effects of the EfE program on the changes in moral cognitions, in line with previous research by Nas and colleagues [47], Van der Velden et al. [38] found more negative attitudes towards antisocial behaviors and lower levels of self-serving cognitive distortions among those students participating to the intervention compared with the control group. However, the effect sizes were small [66]. Instead, with respect to the effects of the EfE program on peer victimization and bullying among high school students, to date, only two quasi-experimental studies [65,67] have been carried out, both in the Spanish context. More in detail, in the earlier study by van der Meulen et al. [65] it was found that the EfE program was partially successful in working on various aspects involved in peers victimization such as in promoting an increase in prosocial behavior by bystanders towards the victims and in reducing some types of bullying and social exclusion behaviors (but only among students whose cognitive distortions reduced). However, there was no overall reduction in victimization and the interpretation of these findings requires several cautions because of the relatively small sample size. Similarly, some years later, van der Meulen et al. [67] found that the EfE yielded positive changes in students’ actions in bullying situations; however, no changes in cognitive distortions and perceived class climate were found after the program’s application.

### 1.3. “Why” and “for Whom” School-Based Anti-Bullying Interventions Could Work Better?

In recent years, the debate among scholars on the effectiveness of school-based programs aimed to prevent and reduce bullying has been focused on the detection of factors accounting for the high variability of their successful [68,69,70,71,72,73,74,75].

The findings from the latest extensive systematic and meta-analytical review of the effectiveness of school-based bullying prevention programs [18] showed that the effectiveness of anti-bullying programs reached an average decrease approximatively of 19–20% for bullying perpetration and 15–16% for bullying victimization outcomes, although with differences across countries and specific interventions.

Given the remarkable variety in the effectiveness of these programs, as a general indication has been suggested by Smith et al. [24] to move from “whether a specific program works or not” (i.e., main effects studies) to uncovering factors that may mediate and/or moderate intervention effectiveness in the sense of exploring “what works, through which mechanisms, for whom, and under what circumstances”.

With regard to the potential mediation mechanisms involved in explaining “why” intervention programs may activate expected behavioral changes, previous meta-analyses focused on the key social-cognitive processes in the explanation of externalizing problem behaviors, that are the “self-exculpatory” cognitive distortions, a general umbrella term that refers to pseudo-justifications and rationalizations for deviant behaviors [76,77,78]. These studies investigating the role of distorted thinking patterns in promoting behavioral changes, as the reduction of externalizing problem behaviors, have not clarified whether treatment success comes about as a consequence of “cognitive restructuring”, i.e., the reframing or correction of cognitive distortions in the treatment which is expected to result in behavioral changes [77,78]. For example, some reviews [79,80] did not reach conclusive empirical evidence that intervention programs designed to address the cognitive attitudes or beliefs impact the subsequent behaviors.

Referring specifically to the EQUIP program, the review by Helmond et al. [80] showed that, within the very small subsample of studies evaluating both cognitive and behavioral outcomes, a significant reduction was found neither in cognitive distortions nor in externalizing problem behaviors. Consequently, the question of whether reducing cognitive distortions could be an effective mediating mechanism for reducing externalizing behaviors remains to be proven.

However, given the widely established link between cognitive distortions and aggressive or antisocial behaviors (e.g., [25,53]), there is good reason to believe that school-based interventions which address biased thinking patterns may, in turn, reduce the involvement in bullying behaviors [37]. In this regard, some studies have shown that cognitive-behavioral interventions, such as the EQUIP program which is specifically focused on the cognitive restructuring, are effective in the subsequent reduction of recidivism rates as well as in improving institutional conduct [62,63]. The study by Liau et al. [63] aimed at investigating if changes in the mediating variables, represented by cognitive distortions and social skills, would be associated with changes in the outcomes related to the treatment, i.e., institutional violations and recidivism rates. The authors found that a significant decrease in cognitive distortions and an increase in social skills promoted fewer serious institutional violations after the intervention, for males and females, respectively.

Referring to individual sociodemographic and psychological characteristics which may improve our knowledge about “for whom” interventions could work better, some studies evidenced that anti-bullying interventions’ efficacy varies depending on age, gender, and the degree of pre-existing symptoms or problematic behaviors, with males [20,31], younger children [81], and children with more severe symptoms and problematic behaviors at baseline [71,82] benefiting more from anti-bullying interventions.

Moreover, over the last decade, a growing number of studies suggested that the effectiveness of interventions varies also depending as a function of the inherent genetic (e.g., [83]), physiological, and psychological [84] characteristics of individuals. In this respect, the Vantage Sensitivity framework [29,30] provides the theoretical basis for the hypothesis that some children may be more likely than others to benefit from intervention because of their heightened sensitivity to positive aspects of the environment. Due to their heightened environmental sensitivity, that is the inherent ability to perceive and process environmental stimuli [29], they could register contextual changes resulting from schoolwide anti-bullying programs more easily and more deeply than other children with lower environmental sensitivity [29,85].

Applied to school-based anti-bullying programs and informed by the theories of Differential Susceptibility [86] and Vantage Sensitivity [29,30], previous studies [31] reported that, although the intervention significantly reduced bullying behaviors and mental health outcomes across the whole sample, individual differences in environmental sensitivity moderated the intervention effects on victimization and internalizing symptoms. More specifically, highly sensitive boys benefited significantly more from the effects of the intervention in reducing both victimization and internalizing symptoms compared to the majority of less sensitive boys. Such findings highlighted that the vantage sensitivity is a useful framework with significant relevance for our understanding of widely observed heterogeneity in treatment response, suggesting that such variability is partly influenced by people’s differing capacity for environmental sensitivity.

Therefore, in light of the empirical evidence discussed above, the need for evidence-based anti-bullying prevention programs, as well as for clarifying the potential mediation and moderation mechanisms involved in explaining the effectiveness of the programs, is highlighted. Furthermore, given the lack of studies having investigated the effects of the EfE program in the Italian school context, the present study was intended to fill the gap in the literature by evaluating the effectiveness of an adapted version of the EfE—implemented as a universal prevention program—in reducing self-serving cognitive distortions and in counteracting the bullying perpetration, also examining “why” and “for whom” the program could be effective.

### 1.4. The Current Study

The main goal of this study was to evaluate the effectiveness of the EfE program [28] on social-cognitive processes (i.e., self-serving cognitive distortions, hereinafter “CDs”) and behavioral (i.e., bullying perpetration) outcome. Since this program has never been tested before in the Italian school context, this is the main novelty of our study. Specifically, given that at the heart of the EfE program is the correction of self-serving CDs and based on the assumption that by altering individual biased thinking patterns, it would be possible to modify antisocial aspects of personality and consequent behaviors [37], we investigated whether the changes in bullying perpetration were mediated through the changes in self-serving moral cognitions after the intervention. Furthermore, guided by the vantage sensitivity framework [29,30] and consistent with the findings of previous studies [31], according to which some individuals may be more likely than others to benefit from intervention due to their sociodemographic and psychological characteristics, we examined whether intervention effects varied depending on adolescents’ gender and individual differences in environmental sensitivity.

As regards the specific hypotheses we made, in line with the aims of the EfE, we expected a significant decrease in the tendency to make self-serving CDs among youth participating in the EfE intervention (i.e., the experimental group) in comparison with youth who did not participate in the EfE (i.e., the control group), and that this effect was stronger for males who were high in environmental sensitivity. Additionally, we expected an indirect intervention effect on the reduction of bullying perpetration through the decrease in the tendency to make self-serving CDs, hypothesizing that this effect was conditional on adolescent gender and individual differences in environmental sensitivity, with highly sensitive males benefiting more from the effects of the EfE, compared with females and less sensitive adolescents [29,85].

## 2. Materials and Methods

### 2.1. Participants

A sample of 354 adolescents—183 males (51.7%; 42.1% middle school students; *M_age_* = 14.99, *SD* = 2.57) and 171 females (48.3%; 46.2% middle school students; *M_age_* = 14.72, *SD* = 2.51)—enrolled in the 7th and 12th grade of three middle schools and three high schools from the provinces of Campania (Italy) were part of a quasi-experimental trial of the EfE program. Among high school students, 30.8% attended a lyceum high school, and 60.2% attended vocational high schools. The overall age of participants ranged from 11 to 21, with a mean age of 14.86 (*SD* = 2.54). Moreover, most of the participants were Italian (98.6%), with 1.4% coming from East Europe (i.e., Albania, Bulgaria, and Ukraine) and from North Africa (i.e., Tunisia and Nigeria).

All selected participants to be involved in the project had to meet the following inclusion criteria: (i) they should attend the second (7th grade) and the fourth (12th grade) year of middle and high schools since these are critical stage for bullying escalation and its progressive decline, respectively; (ii) they should not have participated in the EfE program before; and (iii) their parents should give written consent and the adolescents their own assent to participate in the study.

For each school, four classes were selected by the school staff depending on class teachers’ availability to engage in the program: two classes followed the EfE program (i.e., the experimental group; *n* = 167; 41.3% males; 49.7% middle school students; *M_age_* = 14.62; *SD* = 2.63) while the others followed the normal educational curriculum and did not receive any kind of intervention (i.e., the control group; *n* = 187; 61% males; 39% middle school students; *M_age_* = 15.08; *SD* = 2.43). The total number of classes globally involved in the research project was twenty-four.

By comparing control vs. experimental group on sociodemographic measures, significant differences by gender emerged (χ^2^ (1) = 13.63, *p* < 0.001), with 69 males out 183 (37.7%) compared to 98 females out of 171 (57.3%) belonging to the experimental group. Conversely, no differences by school grade (χ^2^ (1) = 4.07, *p* > 0.05) between the two groups were found.

### 2.2. Study Design and Procedure

A quasi-experimental pre-test/post-test with a control group design was implemented to investigate the effects of the EfE program. The intervention was financed by the Pol.i.s. (Integrated Security Policies) Foundation (for more details about the funding foundation, visit http://fondazionepolis.regione.campania.it/, accessed on 30 May 2022), and was conducted during the 2018/2019 school year. To recruit schools that have been selected from a collaborative network between university and schools in Naples, letters describing the EfE program were sent by the principal investigator of the research project. Six schools joined the project with interest and were further informed about participation possibilities and preconditions. Although each school accepted to participate in the project involving both experimental and control classes, the attribution of classes to the experimental or control condition was made by the school staff rather than through a random selection.

### 2.3. Adaptation of the EfE Intervention

The EfE curriculum was implemented in classroom settings by researchers who were extensively trained before its implementation and received several hours of both individual and group-wise supervision during the intervention period. Prior to the implementation of the intervention, the psychoeducational materials and activities from the EfE curriculum were translated and adapted where this was considered culturally necessary in order to meet the Italian school context. Nevertheless, the deep structure of the EfE program remained unchanged in the Italian version, with only minimal changes which have been made for necessity. The main adjustments concerned the surface structure and the implementation model. For example, due to practical circumstances and school governmental guidelines, the equipment meetings were held only one time per week instead of three times, as advised by the authors of EfE [28]. Thus, we had to reduce the number of meetings from ten to six and, at the same time, we sequentially embedded the three modules within the same meeting. However, according to the course arrangement suggested by the authors [28], we implemented an interrelated agenda teaching across all three components for each equipment meeting to bring out the interrelationships among them, beginning with the Anger Management and Thinking Error Correction, followed by the Social Skills, and then the Social Decision Making component. As a result of these adjustments, the time schedule changed, and the program duration became six weeks instead of ten consecutive week period, with each equipment meeting designed to fit into three hours instead of 45–50 min. Furthermore, other specific modifications to the psychoeducational materials involved changes in the language, pictures included in the materials, and some problem situations (i.e., moral dilemmas) used during the EfE meetings.

The implementation of the EfE program has been structured around the steps described below.

Before starting classroom equipment meetings, a preliminary session was conducted during which trained researchers introduced the EfE curriculum in a positive, motivating manner, communicated the ground rules to be applied during the EfE meetings and taught youth to identify (and even begin to correct) the “thinking errors” (i.e., the CDs) also through a hands-on activity specifically tailored for that purpose (i.e., the “EQUIPPED for Life” game [87]).

After this introduction session, the EfE curriculum was implemented during six classroom meetings structured around the three core components: (i) Anger Management and Thinking Error Correction, to equip youth with skills and strategies, such as self-talking, self-monitoring of emotions and thoughts and thinking ahead about consequences of their acts, to manage anger and to correct self-serving CDs; (ii) Social Skills, to teach youth to constructively solve problems in social situations by learning different social skills (e.g., Overcoming negative peer pressure); and (iii) Social Decision Making, using group discussions around moral values-oriented problem situations to equip youth with mature moral judgment.

As recommended by the authors of EfE [28], several session procedures and techniques were used: working triads, role-playing (not only in the teaching of social skills but also in social decision making and in some anger management sessions), sandwich styles or constructive criticism (in which a critical comment was preceded and followed by supportive ones) and the method called “ask, don’t tell” since the questions stimulated the listener to consider positive, constructive, prosocial alternatives, thus staying focused on the psychoeducational tasks of thinking and acting responsibly.

### 2.4. Data Collection

Approval of the University Institutional Review Board (IRB; protocol code n. 2/2020, dated 13 January 2020) as well as by the School Principal and the class council was obtained for collecting data, which took place through two waves after receiving parents’ written consent and adolescents’ assent, in accordance with the ethical principles of the Italian Association of Psychology (AIP).

Participants of both the control and the experimental group filled out self-report questionnaires, just before starting the program [December 2018, pre-test, Time 1 (T1)] and immediately after [April 2019, post-test, Time 2 (T2)] the end of the implementation of the EfE psychoeducational curriculum. The questionnaires were administered in the classroom, during school time, by trained research assistants. All participants were informed about the voluntary nature of participation and their right to discontinue at any point without penalty.

The American Psychological Association’s ethical standards regarding research with human subjects were followed throughout the research design and implementation.

### 2.5. Measures

#### 2.5.1. Bullying Perpetration

Self-reported bullying perpetration was evaluated at each wave of data collection of the current study by adapting the Florence Bullying and Victimization Scales (FBVSs; [23]). For the purposes of the present study, we only used data about the bullying scale.

Youth were provided with a definition of bullying as intentional, repetitive aggressive behaviors including some sort of power imbalance between those involved, and were asked to indicate, using a 5-point Likert scale (from 1 = ‘Never’ to 5 = ‘Several times a week’), the frequency with which, in the last couple of months, they had exhibited different bullying behaviors as perpetrator, both through direct (i.e., physical, e.g., hitting/kicking, and verbal, e.g., threatening) and indirect (e.g., excluding/ignoring) forms. The scale was composed of the following three subscales, each consisting of three items: physical (e.g., “I hit, kicked, or punched someone”), verbal (e.g., “I threatened someone”), and indirect-relational (e.g., “I excluded someone from activities”) bullying. For each participant, we averaged the 9 items in order to create a composite score of bullying perpetration.

Cronbach’s αs of the overall bullying scale were stable over time, across the two waves of data collection, with αs pre-test = 0.79 and 0.78, and αs post-test = 0.88 and 0.82, for the control and experimental group, respectively.

#### 2.5.2. Self-Serving Cognitive Distortions (CDs)

To evaluate the tendency to make self-serving CDs an Italian validation [88] of the How I think Questionnaire (HIT [48]) was used. At each wave of data collection, participants were asked to indicate their agreement with each of the 39 items using a 6-point Likert scale (from 1 = Disagree strongly to 6 = Agree strongly). Sample items were: “People need to be roughed up once in a while” and “Everybody breaks the law, it’s no big deal”.

The HIT scale was composed of the following four subscales based on Gibbs et al. [42] four-category typology of self-serving CDs: self-centered (9 items), blaming others (10 items), minimizing/mislabeling (9 items), and assuming the worst (11 items). An overall HIT score was computed by averaging the 39 item scores, with a higher score indicating higher levels of CDs.

Cronbach’s αs of the overall CDs scale were stable over time, across the two waves of data collection, with α pre-test = 0.94, and αs post-test = 0.96 and 0.95, for the control and experimental group, respectively.

#### 2.5.3. Environmental Sensitivity

The personality trait of Environmental Sensitivity was measured only at the first wave of data collection by using the Highly Sensitive Child (HSC) scale [89]. The 12 items from which HSC scale was composed were rated by participants on a 5-point Likert scale ranging from 1 (Not at all) to 5 (Extremely). Sample items were: “I notice when small things have changed in my environment” and “Loud noises make me feel uncomfortable”. An overall HSC score was computed by averaging the 12 item scores, with a higher score indicating higher levels of environmental sensitivity.

Cronbach’s αs pre-test were 0.88 and 0.84, for the control and experimental group, respectively.

### 2.6. Analytic Strategy

The data analyzed in this study are available in Supplementary Materials. The statistical analyses were carried out in IBM SPSS statistics version 21 (IBM Corp.; Armonk, NY, USA) and Mplus 8 [90] and have been structured around the steps described below.

#### 2.6.1. Preliminary Analyses

Attrition analyses were performed in order to test if attrition (adolescents with data across the two assessments, i.e., T1 and T2, and those with missing data at T2) was different across groups (control vs. experimental group) and if there were differences in attrition by groups on measures collected at T1.

Before testing our study hypotheses, we verified the comparability (i.e., baseline equivalence) of the two groups (control vs. experimental group), analyzing the differences between them at the pre-test assessment [91]. A Multivariate Analysis of Variance (MANOVA) on the target variables of the EfE program, both on social-cognitive processes (i.e., self-serving CDs) and behavioral (i.e., bullying perpetration) outcome as well as on the personality trait of environmental sensitivity was performed. Based on previous findings revealing significant differences between the control and the experimental group on gender and given that gender- and age-based differences have been observed in CDs (e.g., [37,92]) and bullying behavior (e.g., [93]) in previous research, we included adolescent gender and school grade in the analyses as observed covariates, to control for their potential confounding effects on all study variables.

The partial eta-squared (η^2^_p_) statistic was used to establish the effect size. Levels of η^2^_p_ effect size were interpreted as follows: small (0.01), medium (0.06), and large (0.14) effect size. A *p*-value probability level of <0.05 was adopted for all statistical tests.

#### 2.6.2. The Effectiveness of the EfE Program: The Moderated Mediational Model

**Figure 1:**
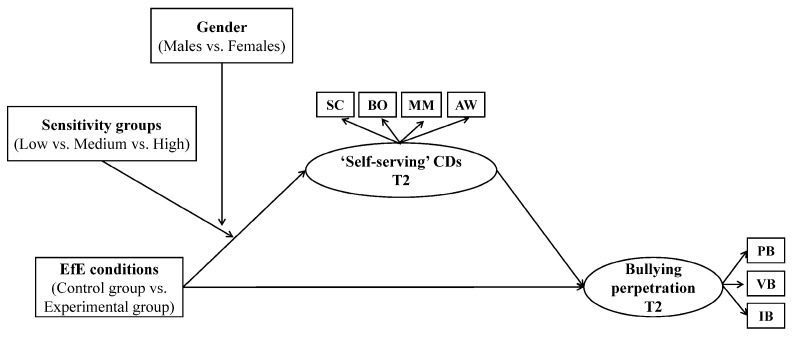
Conceptual representation of the hypothesized longitudinal moderated mediational model. For the sake of clarity, covariates were not shown in the figure. CDs = cognitive distortions; SC = self-centered; BO = blaming others; MM = minimizing/mislabeling; AW = assuming the worst; PB = physical bullying; VB = verbal bullying; IB = indirect-relational bullying.

Subsequently, as regards the mediation and moderation processes involved in explaining “why” and “for whom” the EfE program could be effective, a Structural Equation Modeling (SEM) with latent variables was tested in Mplus 8 [90] using a moderated mediational analysis. More specifically, we investigated whether the change in bullying perpetration was the consequence of the change in CDs derived from the EfE program and if such indirect effects were conditional on adolescent gender (male vs. female) in combination with the sensitivity groups (with low-, medium-, and high-environmental sensitivity). The hypothesized moderated mediational model is graphically shown in Figure 1.

Before testing our moderated mediational model, we firstly identified, within the whole sample and based on the 30/40/30 split approach provided by [89], three distinct “sensitivity” groups: low (bottom 30% of HSC scores), high (top 30% of HSC scores), with the remaining 40% making up the medium sensitive individuals (see Table 1 for the characterization of sensitivity groups); then, concurrently and longitudinally, associations among study variables were performed through Pearson correlations.

In our moderated mediational model, we used as an independent variable the belonging to the control (=0) vs. experimental group (=1), as moderators the gender (1 = male vs. 2 = female) and the belonging to the sensitivity groups (low vs. medium vs. high), that was dummy coded with the group with low sensitivity as the reference group. As dependent variables, the tendency to make self-serving CDs (mediator) and the overall measure of bullying perpetration (outcome) were used. More specifically, for each data wave, given the sample size, the complexity of the model and the number of items and guided by a theory-driven approach, distinct parcels reflecting the respective categories of CDs (i.e., self-centered, blaming others, minimizing/mislabeling, and assuming the worst) and bullying perpetration (i.e., physical, verbal, and indirect or relational bullying) were built through a partial disaggregation approach [94,95] and used as indicators of latent CDs and bullying variables, respectively. The measurement model of the latent variables included in the SEM showed adequate fit to the data (Comparative Fit Index (CFI ≥ 0.95 [96]) and the Root Mean Square Error of Approximation (RMSEA ≤ 0.08 [97]).

All the effects on T2 mediator and outcome were controlled for adolescent school grade and the baseline values (T1), with all variables at T1 allowed to covary. Due to the non-normality of self-serving CDs and bullying perpetration measures (skewness and kurtosis values ranged from 1.33 to 2.04 and from 2.50 to 15.87, respectively) the analyses were performed using the maximum likelihood estimation with robust estimators (MLR). Missing data were handled by using the full-information-maximum-likelihood (FIML) estimation of the parameters. Finally, to evaluate the significance of the moderated mediation effects, we ascertained that the associated confidence intervals did not include zero, thus supporting that the indirect effects via the specific mediator were conditional on the levels of specific moderators (i.e., gender and sensitivity groups).

## 3. Results

### 3.1. Preliminary Analyses: Attrition Rates and Missing Data Analysis

The participation rate was approximately 92% across the two-time points, with 30 (8.5%) of T1 (pre-assessment) participants missed at T2 (post-assessment). The total attrition rate was mainly due to the absence of adolescents from school at assessments. The Little’s test [98] for data missing completely at random (MCAR) in SPSS 21 (IBM Corp.; Armonk, NY, USA) was nonsignificant (χ^2^ (1) = 0.11, *p* > 0.05), indicating no significant difference in attrition rates between the control (20 missed the post-assessment) and experimental group (10 missed the post-assessment). At T2, participants were 324, 169 males (52.2%; 42.6% middle school students; *M_age_* = 14.94, *SD* = 2.56) and 155 females (47.8%; 44.5% middle school students; *M_age_* = 14.81, *SD* = 2.49) ranging in age from 11 to 21 years (*M_age_* = 14.88; *SD* = 2.53).

However, attrition analysis showed significant differences in attrition by groups (control vs. experimental group) on measures collected at T1. Specifically, the interaction between attrition by the experimental group was significant in relation to bullying perpetration (F_(1,350)_ = 7.06, *p* < 0.01, η^2^_p_ = 0.02), with adolescents missing at T2 and belonging to the experimental group reported significantly higher levels of bullying at T1 than those who had data at all assessments. Differently, no significant differences in attrition by groups emerged for self-serving CDs (F_(1,350)_ = 0.65, *p* > 0.05, η^2^_p_ = 0.00).

### 3.2. Baseline Equivalence: Comparison between Control and Experimental Group before EfE Intervention

Preliminarily, a MANOVA was conducted to investigate group (0 = control vs. 1 = experimental group) differences with respect to the baseline measures also controlling for the potential confounding effects of adolescent gender (1 = males vs. 2 = females) and school grade (1 = middle vs. 2 = high school students).

There was only a significant main effect of gender (Wilks’s λ = 0.92; F_(3,314)_ = 9.09, *p* < 0.001, η^2^_p_ = 0.08) on baseline measures, while no significant main effects of school grade (Wilks’s λ = 0.98; F_(3,314)_ = 1.98, *p* > 0.05, η^2^_p_ = 0.02) and EfE conditions (Wilks’s λ = 0.99; F_(3,314)_ = 1.22, *p* > 0.05, η^2^_p_ = 0.01) were found. Moreover, any interaction effects between gender and school grade (Wilks’s λ = 0.98; F_(3,314)_ = 1.73, *p* > 0.05, η^2^_p_ = 0.02), as well as between such variables with EfE conditions (Wilks’s λ = 0.99; Fs_(3,314)_ = 0.45 and 1.43, *p* > 0.05, η^2^_p_s = 0.00 and 0.01, for gender and school grade, respectively) emerged. As Table 2 shows, at the pre-test assessment no significant differences were found between control and experimental group on the tendency to make self-serving CDs as well as on the involvement in bullying perpetration, and regarding individual trait of environmental sensitivity. Differently, looking at the gender-related effects, significant differences were found with males scoring higher in the tendency to make self-serving CDs as well as in perpetrating bullying, and lower in environmental sensitivity compared to females.

### 3.3. Correlations between Study’s Variables

Descriptive statistics and zero-order correlations between all study’s variables are shown in Table 3. As can be observed, all variables were significantly intercorrelated with each other, both in the control and experimental group, both concurrently, within the same time point and, longitudinally, across time. High school students belonging to the experimental group reported significant higher levels of CDs at T2.

### 3.4. Moderated-Mediational Process Model: Testing the Direct, Indirect, and Conditional Effects of EfE Program

Results of the moderated mediational SEM evaluating whether the EfE program was associated with the reduction of bullying perpetration through the decrease of self-serving CDs over time, and if the hypothesized indirect effect of the EfE program was conditioned by gender and by the belonging to the distinct sensitivity groups, controlling for the potential confounding effects of adolescent school grade (1 = middle vs. 2 = high school students) and for prior (baseline) measures of each variable, are reported in Table 4.

**Figure 2:**
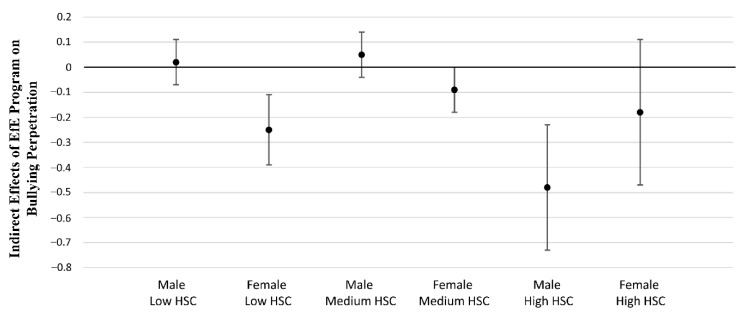
Plot of the indirect effects of EfE program on bullying perpetration through self-serving cognitive distortions conditional on gender and sensitivity groups with low, medium, and high scores on the Highly Sensitive Child (HSC) scale.

As shown in Table 4, the two-way and three-way interaction terms between the EfE conditions (= belonging to the experimental group) and sensitivity groups (= belonging to the group with high levels of environmental sensitivity) as well as between such variables with gender groups (= males) on the change of self-serving CDs were significant, controlling for the baseline (T1) measure of CDs. The analysis of the indirect effects moderated by sensitivity groups and gender showed a significant moderated mediation effect for males belonging to the high (vs. low) sensitivity group. The conditional indirect effect is plotted in Figure 2. Overall, these results suggest that participating in the EfE program led to a decrease in bullying perpetration by equipping youth with skills for correcting their “thinking errors” or self-serving CDs, with highly sensitive males benefiting significantly more from the effects of the intervention than females and those with lower levels of environmental sensitivity.

## 4. Discussion

The present study represents the first attempt to evaluate the effectiveness of the EfE program—implemented as a universal prevention program [28] in the Italian school context—in correcting adolescents’ use of self-serving CDs as well as in counteracting the bullying perpetration, by using a quasi-experimental pre-test/post-test with control group design. Furthermore, following the general indication to move from the investigation of “whether a specific program works or not” to uncovering “what works, through which mechanisms, for whom, and under what circumstances” [24], we tried to clarify the mediation and moderation mechanisms involved in explaining “why” and “for whom” the EfE program may work effectively.

Our results supported the effectiveness of the EfE program in correcting adolescents’ “thinking errors” or self-serving CDs which, in turn, was associated with the reduction of bullying perpetration, but only among highly sensitive males. Conversely, no independent effect of the EfE program emerged, neither on social-cognitive processes (i.e., self-serving CDs) nor on the behavioral outcome (i.e., bullying perpetration).

These findings seem to be in line with most of the previous research, which established that the EQUIP program is successful in promoting changes in social-cognitive processes, such as self-serving CDs, both in juvenile correctional facilities (e.g., [47,64]) and school settings (e.g., [32,38,65]). In addition, informed by the theories of differential susceptibility [86] and vantage sensitivity [29,30], our results are consistent with previous studies showing the moderating role of environmental sensitivity in predicting children’s treatment response to established psychological intervention focused on reducing both internalizing and externalizing problems [31,85]. More specifically, in line with the study by Nocentini et al. [31], we found that the moderating effects of environmental sensitivity varied depending on gender, with more pronounced effects in males than in females. Based on findings from previous studies highlighting gender-based differences in CDs (e.g., [37,92]), a reasonable justification for the moderating role of gender might be that highly sensitive males could benefit directly from a treatment-induced reduction in CDs and be generally more perceptive of positive changes in the school and classroom context after the intervention. Furthermore, behind the “advantage” of environmental sensitivity in enhancing the treatment response, it could be possible that highly sensitive males could be more perceptive and aware of their surroundings, more likely to register program-induced improvements, such as the reduction of self-serving CDs, more easily and more deeply [99,100], thus leading to better internalization of the acquired cognitive thinking patterns than females and less sensitive youth (e.g., [85]). In this regard, consistent with empirical evidence from neuroimaging and genetic studies (e.g., [101,102]), the greater treatment response of highly sensitive individuals may be due to their specific neural and genetic characteristics that contribute to brain activities related to a deeper processing of environmental influences, greater ability to direct attention heightened, and reward sensitivity [30].

Moving towards the mediation mechanisms involved in explaining “why” the EfE program may activate expected behavioral changes, we found that among males highly sensitive to environmental influences, the EfE program was effective in reducing the perpetration of school bullying through the decrease of self-serving CDs, whose correction is at the heart of the EfE psychoeducational curriculum [32]. Overall, this result is consistent with the social-cognitive approaches (e.g., [50,59]) and in line with the suggestions provided by previous studies highlighting the role of social-cognitive processes, such as the self-serving CDs, in promoting behavioral changes, as the reduction of externalizing problem behaviors (see [76,77,78]).

To our knowledge, only two studies [65,67] have specifically investigated whether the EfE program might be useful to work on various aspects involved in peer victimization in the school context finding a slight decrease in bullying as perceived by the students themselves during their daily school life. However, although these results provide some support for the idea of increased bullying awareness among students, really behavioral changes in aggressive behaviors among the bullies have not been investigated.

Moreover, the lack of direct effects of the EfE program in reducing school bullying perpetration we found could be understood in light of a previous meta-analytic review [103] specifically focused on the effects of the program on more serious antisocial acts or recidivism rates. As argued by the authors, although most of the previous studies have well documented the efficacy of the program in addressing the main youthful offender’s problematic tendencies or limitations (in terms of sociomoral developmental delays or deficiencies), its overall effect on recidivism was not significant. For example, previous attempts [63,64] to implement the EQUIP with offenders reached similar results highlighting that the program fails in reducing the speed or seriousness of reoffending [64] as well as in decreasing self-reported aggression and delinquency [63]. Conversely, other studies have found a substantial reduction of recidivism rates after the intervention [61,62,63].

However, with modest magnitude, the moderated mediation effect of self-serving CDs on the reduction of bullying perpetration seems to be consistent with previous similar research aimed at investigating whether cognitive-behavioral interventions are specifically designed to address whether the cognitive attitudes or beliefs may impact the subsequent behaviors. For example, previous studies using the original treatment version of the EQUIP program with juvenile [64] and adult [62,63] offenders, found that the gains in social-cognitive processes, as measured by the reduction in the level of CDs, were significantly related to fewer serious institutional infractions as well as to the speed of recidivism with a longer time interval latency before reoffending [63,64] and lower recidivism rates [62].

In addition, our findings seem to make sense in light of the structuring of the EfE program, which is specifically focused on the cognitive restructuring, i.e., the reframing or correction of CDs, which is expected to result in behavioral changes [77,78]. Most of the activities provided in the *Anger Management* or *Thinking Error Correction* component of EfE program were devoted to working intensively to equip youth with skills, such as self-monitoring of emotions and thoughts and thinking ahead, improving anger management, and correcting self-serving CDs, that can help them to refute the rationalizations or justifications that block or neutralize their empathy for actual or prospective victims [28]. Given that moral cognitive processes, as the self-serving CDs, represent a key factor for motivating aggressive behaviors among peers (e.g., [25]), such skills have been found to enhance the possibility to inhibit youths’ engagement in bullying behaviors.

Summarizing, the present study supports the effectiveness of the EfE program in equipping youth to think and act (more) responsibly. Indeed, by equipping youth with skills for correcting their “thinking errors” or self-serving CDs when interpreting social events, the EfE program led to positive behavioral changes, specifically a more responsible way of (inter)acting with peers with enhancer effects for males highly sensitive to environmental influences. Therefore, our findings suggest that the social-cognitive approaches (e.g., [50,59]) together with the vantage sensitivity are useful frameworks with significant relevance for our understanding of “why” and “for whom” the intervention could work better to counteract bullying in the school context.

### Limitations and Future Directions

This study has some limitations that need to be acknowledged. As a first methodological limitation, the intervention was implemented at the class-level with all students in the class participating and the assignment to one or another condition was not randomized (see the procedure section). For these reasons, as suggested by Gottfredson et al. [91], both in preliminary analyses and in all the subsequent analyses, we tested for the baseline differences between the two groups and controlled the possible effects of sampling (i.e., the effects of the program were controlled for the baseline values of the target variables). Although the method we used can be considered at least acceptable, it would be desirable to replicate our findings using Randomized Control Trials (RCTs) designs.

Another potential caveat related to the program implementation that may have influenced the program-related outcomes evaluation and that we have not thoroughly investigated concerns the extent to which the program procedures are carried out as intended (i.e., the program integrity [104]). It should be noted that, our study, represent the first attempt to adapt the EfE program in the Italian school context. Although the deep structure of the EfE program remained unchanged, the lower intensity or frequency and the shorter duration of the program may have influenced the outcomes achieved, as found in previous studies (e.g., [63]). Future studies on the effectiveness of the EfE program could benefit more from the inclusion of objective measures of program integrity.

In addition, due to the sudden spread of the COVID-19 pandemic, we were unable to carry out “at least one long-term follow-up at an appropriate interval beyond the end of the intervention […]” [91] (p. 897). Future longitudinal studies are needed to shed light on whether the EfE program might be effective to promote long-term cognitive and behavioral changes.

Additionally, all the measures were self-reported, and they could be subject to social desirability bias. Indeed, although adolescents have direct knowledge of their own experiences and behaviors, it is widely known (e.g., [105]) that they are more careful about their social image than other age groups and may be unlikely to report attitudes or behaviors that display them in a negative light. Thus, regarding the tendency to make self-serving CDs as well as to perpetrate bullying, in future research, observational or multiple-source data (e.g., peers’ and teachers’ reports), could be used to provide more objective information. Related to this issue, another possible limitation concerns the lack of information about further relevant program-related variables; indeed, in our study, we only focused on the effects of the program on self-serving CDs and bullying perpetration. Future research should also include other relevant components of the program, such as the social skills and the moral judgment, as well as also more qualitative data from students, which could shed more light on their perceptions of the group dynamics before, during, and after the program and of changes of their own and their classmates’ cognitions and behaviors.

A final methodological limitation is related to the generalizability of our results, as the study included a sample from a limited geographical area in Southern Italy. Since this is the first study carried out in Italy, and being aware that multiple factors, including culture-specific beliefs and values, influence an individual’s cognitions and behaviors [106], additional studies involving adolescents populations from other, possibly differing, cultural contexts are needed to generalize the effectiveness of the EfE program.

## 5. Conclusions and Policy Implications

Notwithstanding the limitations discussed above, this study integrates previous knowledge and provides some relevant suggestions to researchers and practitioners for implementing appropriate interventions aimed at reducing adolescents’ involvement in bullying behaviors.

Guided by a clear theory of causal mechanisms related to our behavioral outcome, represented by the social-cognitive approaches (e.g., [50,59]), the current study highlighted the potential mechanisms involved in explaining “why” and “for whom” the EfE program could work better to promote the expected changes. Specifically, we found that the EfE program was effective in counteracting the bullying perpetration through the reduction of self-serving CDs, which are more likely to decrease among males higher in sensitivity to environmental influences.

In sum, it can be concluded that, as originally intended [32], the EfE has the potential to change cognitions and problem behaviors by equipping youth to think and act more responsibly. As the correction of “thinking errors” or self-serving CDs has been found to play a crucial role in counteracting the bullying perpetration among youth participating to the program, our study points to the benefit of school-based approaches that target the strengthening of youths’ moral cognition and that makes cognitive restructuring techniques (i.e., the reframing or correction of CDs [77,78]) one of its main strengths. Moreover, the current study provided evidence of the enhancer effect of environmental sensitivity in improving adolescents’ response to intervention, thus suggesting that the implementation of anti-bullying intervention in the school context could benefit from the early identification by teachers of those students higher in environmental sensitivity. Indeed, while highly sensitive students may require shorter or lower intensity intervention programs, vantage-resistant individuals may require more intensive intervention approaches or rather alternative types of treatment to benefit from them.

Overall, in terms of practical implications in the educational field, our findings provide some suggestions for preventing the detrimental outcomes associated with school bullying experiences, on both perpetrators, victims, and on the school community as a whole. According to the multi-modal approach of the EfE program, the current study calls specific attention to the need to support adolescents in developing skills that could help them to successfully cope with peer-related violence, including emotion self-regulatory abilities (particularly relating to anger), social problem-solving skills, and moral education.

Based on positive peer culture, in which adolescents feel responsible for each other and help one another, it could be useful to implement the EfE program in other school contexts where it is expected to have a great public impact given that it promotes, in the long term, the development of a nonviolent and law-abiding culture, which represents the crucial condition for ensuring success in preventing and reducing bullying phenomena among youth in their daily school life.

## Figures and Tables

**Table 1:** Identification and sociodemographic characteristics of sensitivity groups.

	Low HSC ≤30% HSC		Medium HSC 30% < HSC ≥ 70%		High HSC >70% HSC	
	Males	Females	Males	Females	Males	Females
Total sample *n* = 324	64 37.9%	32 20.6%	72 42.6%	70 45.2%	33 19.5%	53 34.2%
Control group *n* = 167	39 37.1%	12 19.4%	49 46.7%	28 45.2%	17 16.2%	22 35.5%
Experimental group *n* = 157	25 39.1%	20 21.5%	23 35.9%	42 45.2%	16 25%	31 33.3%

Note: Reported number of subjects belonging to the control and experimental groups refers to those who participated in both waves of data collection. Reported percentages are valid percentages.

**Table 2:** Descriptive statistics and ANOVAs results by groups (control vs. experimental group), gender (males vs. females), and school grade (middle vs. high school students) in self-serving cognitive distortions (CDs), bullying perpetration, and environmental sensitivity, before EfE.

Measures	EfE Conditions		Gender		School Grade		Groups Differences		Gender Differences		School Grade Differences	
	Control Group (*n* = 167)	Experimental Group (*n* = 157)	Males (*n* = 169)	Females (*n* = 155)	Middle (*n* = 141)	High (*n* = 183)						
	Means (SDs)						F_(1,316)_	η^2^_p_	F_(1,316)_	η^2^_p_	F_(1,316)_	η^2^_p_
Self-serving CDs	2.08 (0.75)	2.09 (0.76)	2.17 (0.76)	1.99 (0.74)	2.09 (0.84)	2.08 (0.68)	0.29	0.00	3.85 *	0.01	0.07	0.00
Bullying perpetration	1.36 (0.51)	1.26 (0.39)	1.39 (0.53)	1.24 (0.35)	1.32 (0.45)	1.21 (0.33)	2.10	0.01	5.99 *	0.02	3.62	0.01
Environmental sensitivity	3.04 (0.86)	3.12 (0.81)	2.91 (0.87)	3.26 (0.76)	3.01 (0.95)	3.13 (0.73)	0.00	0.00	13.39 ***	0.04	1.76	0.01

Note: For the sake of simplicity, nonsignificant interaction effects are omitted. * *p* < 0.05, *** *p* < 0.001; η^2^_p_ = partial eta-squared effect size.

**Table 3:** Correlations between study’s variables, means (M) and standard deviations (SDs).

Measures	1	2	3	4	5
1. School grade (High)	1	−0.06	−0.08	−0.07	−0.15
2. T1 Self-serving CDs	0.06	1	0.62 ***	0.47 ***	0.40 ***
3. T2 Self-serving CDs	0.16 *	0.61 ***	1	0.33 ***	0.46 ***
4. T1 Bullying perpetration	−0.14	0.35 ***	0.30 ***	1	0.52 ***
5. T2 Bullying perpetration	−0.09	0.34 ***	0.42 ***	0.35 ***	1
M	-	2.08 (2.09)	1.92 (1.78)	1.36 (1.26)	1.32 (1.25)
SDs	-	0.75 (0.76)	0.78 (0.73)	0.51 (0.39)	0.55 (0.42)
Range	-	1–6		1–5	

Note: School grade was coded 1 = middle and 2 = high school; CDs = cognitive distortions. Data for the control group appear above the diagonal and data for the experimental group appear below the diagonal. Values in brackets refer to the experimental group. * *p* < 0.05, *** *p* < 0.001.

**Table 4:** Direct and indirect effects of the EfE Program on bullying perpetration through self-serving cognitive distortions (CDs) conditional on gender and sensitivity groups with low, medium, and high scores on the Highly Sensitive Child (HSC) scale.

Unique and Interactive Effects							
Predictors		Self-Serving CDs (T2)			Bullying Perpetration (T2)		
		B	SE	95% C.I.	B	SE	95% C.I.s
EfE conditions (Control vs. Experimental group)		4.54	3.56	[−2.44, 11.53]	−0.00	0.13	[−0.26, 0.26]
Sensitivity groups (Low vs. Medium HSC)		3.96	3.13	[−2.18, 10.10]	−0.04	0.16	[−0.34, 0.27]
Sensitivity groups (Low vs. High HSC)		9.87 *	3.88	[2.26, 17.47]	−0.02	0.18	[−0.38, 0.33]
Gender (Male vs. Female)		1.50	1.87	[−2.18, 5.17]	−0.12	0.14	[−0.40, 0.16]
Experimental group × Medium HSC		−1.62	4.78	[−10.99, 7.75]	-	-	-
Experimental group × High HSC		−16.90 **	5.57	[−27.82, −5.98]	-	-	-
Experimental group × Gender		−4.28	2.53	[−9.24, 0.68]	-	-	-
Medium HSC × Gender		−2.95	2.30	[−7.46, 1.57]	-	-	-
High HSC × Gender		−6.13 *	2.62	[−11.26, −0.99]	-	-	-
Experimental group × Medium HSC × Gender		2.10	3.22	[−4.21, 8.41]	-	-	-
Experimental group × High HSC × Gender		10.86 **	3.59	[3.82, 17.90]	-	-	-
Self-serving CDs (T2)		-	-	-	0.06 ***	0.01	[0.04, 0.08]
**Conditional Indirect Effects of the EfE Program at Different Values of Gender and Sensitivity Groups (Moderators)**							
Self-serving CDs (T2–Mediator)	Males with Low HSC	-	-	-	0.02	0.09	[−0.13, 0.17]
	Females with Low HSC	-	-	-	−0.25	0.14	[−0.47, 0.03]
	Males with Medium HSC	-	-	-	0.05	0.09	[−0.10, 0.19]
	Females with Medium HSC	-	-	-	−0.09	0.09	[−0.23, 0.05]
	Males with High HSC	-	-	-	−0.48 *	0.25	[−0.88, −0.07]
	Females with High HSC	-	-	-	−0.18	0.29	[−0.66, 0.30]

Note: Both for medium and highly sensitive subjects the reference group was that with low sensitivity. B = Unstandardized estimates; SE = Standard error; C.I.s = Confidence intervals; For the sake of simplicity, the relations with control variables are omitted. * *p* < 0.05, ** *p* < 0.01, *** *p* < 0.001.

## Data Availability

The data presented in this study are available in Supplementary Materials.

## Supplementary Materials

The following supporting information can be downloaded at: https://www.mdpi.com/article/10.3390/ejihpe12070060/s1, Supplementary materials only include the dataset supporting the findings of the study. This has been indicated in the main text (in the Analytic Strategy section) and in the data availability statement.
